# Stress among general practitioners of Kwa-Dukuza, Kwa-Zulu Natal

**DOI:** 10.4102/phcfm.v1i1.39

**Published:** 2009-08-04

**Authors:** Indiran Govender, Gina Joubert, Stefanus D.W. Oosthuizen

**Affiliations:** 1 Department of Family Medicine, University of Limpopo, South Africa; 2 Department of Biostatistics, University of the Free State, South Africa; 3 Department of Community Health, University of the Free State, South Africa

**Keywords:** general practitioners, burnout, depression, extended working hours, stress

## Abstract

**Background:**

Stress and burnout are prevalent among the caring professionals, including doctors and nurses. The work-related stress rate among the general working population is 18% whilst among doctors it is around 28%. Stress in general practitioners (GPs) can result in multiple negative consequences. Detecting stress early may have positive outcomes for doctors, their families and the people they care for at their practice.

**Method:**

A cross-sectional, descriptive study using a self-administered, standardised questionnaire (12-item General Health Questionnaire [GHC]) was performed on the 30 general practitioners in Kwa-Dukuza. Confidentiality and anonymity were maintained.

**Results:**

26 of the 30 GPs (87%) responded to the survey. 10 GPs (38%) were stressed as per the GHQ, six of whom were severely stressed. 22 reported that they felt stressed at work (subjectively).

**Conclusion:**

The results indicated that stress among Kwa-Dukuza GPs is slightly higher (38%) than found in other studies that indicate a prevalence of 28% among doctors.

## INTRODUCTION

Most people spend most of their waking hours at work.^1^ This world of work may generate in many people emotional, mental and/or physical disturbances that should be prevented and/or may need treatment. Thus, the quality and nature of people's experience at work may result in major health implications.^1^

Demanding or frustrating work or an inability to cope with stressors at work can cause various short- and long-term effects. These effects can be physical, mental, physiological (increased blood pressure, cardiovascular diseases), psychological/emotional (tenseness, anxiety disorders) or behavioural (alcohol abuse).^1^ This specific response of the body to all non-specific demands is called stress.^2^.

Are doctors any different from the general working population? It may be surprising to lay people that not every doctor is happy in his or her professional life. Stress and burnout (which is an extreme response to stress) among the caring professionals, including doctors and nurses, are among the highest of all professions.^3, 4^ Stress among the general working population is around 18% whilst stress among doctors is 28%.^5^

Stress among doctors is not a modern phenomenon. As reported by Ross and Deverell, Sir William Osler in the 19^th^ century said, ‘To each one of you the practice of medicine will be very much as you are to it – to one a worry, a care, a perpetual annoyance; to another, a daily joy and a life of as much happiness and usefulness as can well fall to the happiest man’.^6^

Stress among doctors not only comes from external factors called stressors but is also internally generated by doctors’ hopes, fears, expectations and beliefs.^2^ Therefore, Osler's statement is appropriate and what is stressful to one doctor will be a challenge to another, depending on their perception of their situation and their ability to cope with that circumstance.^2^

The four factors that may result in stress are external demands (stressors), internal needs and values, personal coping resources and external resources or support.^2^

Burnout is described as a state of physical, emotional and mental exhaustion caused by excessive and/or prolonged stress.^7^ Burnout is so common that the American Joint Commission on Accreditation of Healthcare Organizations (JCAHO) insists that all hospitals have processes to improve physician well-being at work.^3^

The modern medical workplace is a stressful place.^6^ It is a complex environment where doctors have to continually learn new skills.^6^ Stress also arises from long hours, constantly caring for ill people, facing death and dying among people whom family physicians have come to know and knowing that their occupation carries enormous responsibility and that people's lives depend on them.^4, 5, 6^ Other factors are fatigue; high demands on time, thus interfering with doctors’ other responsibilities; work conflicting with doctors’ personal lives; dealing with emergencies; uncertainty and error; patient consumerism; increasing demands from patients; financial pressures of balancing a business and helping people; information overload; administration; and personality factors.^4, 5, 6^

It has been emphasised that stress is highest among those doctors caring for terminally and chronically ill patients.^8^ General practitioners (GPs) are frequently the carers of this group of patients where the prognosis is often poor. They also have to deal with stress from litigation threats and medical aid and government intervention, which adversely affect the autonomy of the GP. Olkinuora et al. confirm that the occurrence of stress symptoms is high among GPs.^5^

This stress in GPs results in high rates of marital problems, which sometimes end in divorce;^5, 8^ physical illness; social isolation;^5, 8^ decreasing satisfaction with work;^5, 8^ suicide;^9^ substance abuse; and depression.^10^

Medical decisions are impaired in the stressed doctor, leading to substandard care or outcomes that are even more disastrous for the patient. Detecting stress early may have positive outcomes for doctors, their families and the people they care for at their practice.^5^

Kwa-Dukuza is located on the north coast of Kwa-Zulu Natal, South Africa. This town is approximately 90 kilometres north of Durban. There are 31 GPs in private practice in Kwa-Dukuza. These doctors see both patients who are on medical aid and those who are not. Only one practice has two partners while the rest are solo practices with doctors working alone every day, sometimes working on Sundays and attending to after-hours emergency calls from their patients. There are divorced doctors in the area.

This study was discussed with the local Independent Practitioners Association (IPA) and the results will be presented to all members of the IPA at a Continuing Medical Education (CME) meeting. The IPA will then decide from the results whether there is a need for weekend workshops with experts in the field of stress management. The researcher's discussion with the IPA concerning this has received much interest and the organisation is willing to spend its funds on this type of workshop. This will contribute to the occupational health of doctors from Kwa-Dukuza.

## METHOD

To determine the prevalence and level of stress among the GPs of Kwa-Dukuza, a cross-sectional, descriptive study using a self-administered questionnaire was carried out.

All GPs of Kwa-Dukuza except Indiran Govender were part of the study population. There were 30 Gps in the study population as at August 2007, of which three were female. This number was the same for the Lancet Laboratory and Kwa-Dukuza Independent Practitioners lists. All the GPs had an excellent comprehension of English.

A pilot study, mainly to assess the questionnaire, was carried out. Five GPs who were located in another town (Richards Bay) were selected for the pilot study. This was to ensure that all the GPs in Kwa-Dukuza could take part in the study and also to ensure that the results were not influenced by the pilot study doctors’ discussing the study with their colleagues of Kwa-Dukuza before the actual study was conducted. The questionnaire worked well and doctors took between five to seven minutes to complete the questionnaire.

All GPs were telephoned personally by the researcher prior to the study, informing them of the study and requesting their participation. Confidentiality was maintained. Doctors were also informed of the study at the CME meetings where most doctors meet on a social level. The questionnaires were distributed by the Lancet courier on a Monday (7 January 2008) and collected up to the end of the fourth week post distribution (1 February 2008). This took longer than anticipated as many doctors only sent in completed questionnaires after three weeks. All doctors were reminded telephonically to send in completed questionnaires. Doctors were also reminded of the research at the weekly evening CME meetings. The completed questionnaires were placed in a box at the researcher's practice by the Lancet courier.

The measurement instrument was a standardised, anonymous questionnaire with voluntary participation. The 12-question General Health Questionnaire (GHQ) was used to assess whether the GPs were stressed. The questionnaires were in English. The GHQ is used by researchers in various fields, including occupational health, and by clinicians to screen for psychiatric disorders.^11^ It is a short questionnaire with 12 questions with three-point Likert scale responses. Ease of completion was important since the doctors are usually busy and may not have taken the time to complete a long questionnaire. It is a validated questionnaire with high sensitivity that has been widely used to screen populations for stress.^11^ The GHQ-12 yielded an overall total score.^12^ The score was then used to gauge whether each doctor was not stressed (total less than 16), was stressed (total 16–20) or had severe psychological stress (total more than 20).^12^ The questionnaire was modified slightly to exclude the name of the doctor and to include age group, hours per working week, length of time in GP practice and a general self-perception of whether the doctor was stressed or not.

The cover page of the questionnaire explained the purpose of the study. This page also contained the contact details of the researcher.

The response rate was low by the end of the first week; the doctors were then personally telephoned on the Monday of the second week and reminded of the study and requested to complete the questionnaire if they had not already done so.

The questionnaires were anonymous with no information on the questionnaire that could identify the doctor. The distribution and collection of the questionnaires was anonymous. This was done to improve the truthfulness of the responses.

Ethical approval of the study was obtained from the Ethics Committee of the Faculty of Health Sciences of the University of the Free State.

## RESULTS

26 of the 30 GPs (87%) responded to the survey. The respondents answered all questions.

Table 1 shows representation of doctors from all age groups with the majority of doctors in the below-40-years age group. A large proportion of the doctors work more than the average 40-hour week. The responding GPs have differing years of experience. More than half of the responding GPs receive after-hours calls.

**TABLE 1:** Age group, average hours worked by GP per week, length of GP practice and afterhour calls

	FREQUENCY	PERCENTAGE
**AGE GROUP**		
28–39	14	54
40–49	6	23
50–59	5	19
> 60	1	4
**AVERAGE HOURS WORKED PER WEEK**		
30–40	7	27
41–50	9	34
51–60	8	31
61–70	2	8
**LENGTH OF PRACTICE**		
< 5 years	6	23
5–9.9 years	5	19
10–15 years	7	27
> 15 years	8	31
**AFTER-HOUR CALLS**		
No	10	38
Yes	16	62

There is a wide variation in the number of patients each GP sees per day. The median number of patients seen per day is 30, with a range of 12 to 60. Half of the GPs usually consult with 30 to 50 patients per day.

Table 2 shows that 22 doctors (85%) reported that they felt stressed at work (subjectively).

**TABLE 2:** Subjective feeling of being stressed at work

FEELING OF BEING STRESSED	FREQUENCY	PERCENTAGE
No	4	45
Yes	22	85

Table 3 represents the responses to the individual questions as they appear in the GHQ-12.

**TABLE 3:** Results of the GHQ-12 questions

	FREQUENCY	PERCENTAGE
**ABILITY TO CONCENTRATE**		
Better than usual (0)*	4	15
Same as usual (1)	16	62
Less than usual (2)	5	19
Much less than usual (3)	1	4
**LOSS OF SLEEP DUE TO WORRY**		
Not at all (0)	2	8
No more than usual (1)	15	57
Rather more than usual (2)	7	27
Much more than usual (3)	2	8
**PERCEPTION OF PLAYING A USEFUL PART IN WORK**		
More so than usual (0)	6	23
Same as usual (1)	11	42
Less so than usual (2)	6	23
Much less so than usual (3)	3	12
**CAPABLE OF MAKING DECISIONS**		
More so than usual (0)	5	19
Same as usual (1)	13	50
Less so than usual (2)	6	23
Much less so than usual (3)	2	8
**FEELING CONSTANTLY UNDER STRAIN**		
Not at all (0)	2	8
No more than usual (1)	7	27
Rather more than usual (2)	14	54
Much more than usual (3)	3	11
**GPS’ PERCEPTION OF THEIR ABILITY TO OVERCOME THEIR DIFFICULTIES**		
Not at all (0)	4	15
No more than usual (1)	11	42
Rather more than usual (2)	8	31
Much more than usual (3)	3	12
**ABILITY TO ENJOY NORMAL DAY-TO-DAY ACTIVITIES**		
More so than usual (0)	1	4
Same as usual (1)	12	46
Less so than usual (2)	11	42
Much less so than usual (3)	2	8
**GPS’ ABILITY TO FACE UP TO THEIR PROBLEMS**		
More so than usual (0)	3	11
Same as usual (1)	14	54
Less so than usual (2)	8	31
Much less so than usual (3)	1	4
**BEEN FEELING UNHAPPY OR DEPRESSED?**		
Not at all (0)	4	15
No more than usual (1)	12	46
Rather more than usual (2)	9	35
Much more than usual (3)	1	4
**BEEN LOSING CONFIDENCE IN YOURSELF?**		
Not at all (0)	9	35
No more than usual (1)	9	35
Rather more than usual (2)	7	27
Much more than usual (3)	1	3
**BEEN THINKING OF YOURSELF AS A WORTHLESS PERSON?**		
Not at all (0)	13	50
No more than usual (1)	9	35
Rather more than usual (2)	3	11
Much more than usual (3)	1	4
**BEEN FEELING REASONABLY HAPPY, ALL THINGS CONSIDERED?**		
More so than usual (0)	2	8
Same as usual (1)	16	61
Less so than usual (2)	7	27
Much less so than usual (3)	1	4

* The number in brackets denotes scores given as per Likert scale

10 GPs were stressed as per the GHQ (38%), six of whom were severely distressed (see Table 4).

**TABLE 4:** Summary of totals from the objective GHQ-12 questions

STRESS LEVEL	FREQUENCY	PERCENTAGE
Severely stressed	6	23
Stressed	4	15
Not stressed	16	62

Table 5 shows that eight of the 10 doctors subjectively stressed were also stressed according to the GHQ.

**TABLE 5:** Comparison of doctors stressed as per GHQ scoring and their subjective perception of stress

	NUMBER OF RESPONDENTS
Subjectively stressed	22
Objectively stressed	10
Subjectively and objectively stressed	8

## DISCUSSION

The high response rate of 87% may have been helped by the methodology, which ensured anonymity and confidentiality. A particular doctor could not have been easily identified. Any means to identify the particular doctor in this small town may have led to a low response rate and thus results that would not have been valid. The limitation of this methodology is that those who are stressed may not be easily identified for further management. This, however, was not the aim of the study. The response rate was also improved by personal telephone calls by the researcher and mention of the study at the CME gatherings before and during the study. The high response rate improves the validity of this study.

Those doctors who are more stressed could have been more likely to complete the questionnaire, as an expression of help-seeking behaviour. Thus, more doctors who are stressed may have responded to the study. This may then distort the true picture. On the other hand, doctors who are not stressed may also have completed the questionnaire. There was a sufficiently high response rate of more than 80% to overcome this response bias.

The results of this study may not be generalisable to doctors in other fields of practice or to GPs in other geographic areas of practice since this study was limited to the GPs of Kwa-Dukuza. This small study population of GPs limits the results to this population, limiting objectivity and generalisability.

The number of the respondents was small and thus no clear relationship could be established between stress and age group, number of years working as a GP, after-hours work or number of hours worked per day.

The results regarding the age group (in Table 1) of the GPs of Kwa-Dukuza show a representation of doctors from all age groups. This is in keeping with the age group of the doctors in the town. The majority of respondents were in the age group 28 to 39 years (54%). Thus work-related stress may also affect doctors who have not been in practice for many years. Work-related stress seems to be prevalent in all age groups in this sample of doctors. This is in keeping with the literature that demonstrates that modern medical practice is extremely demanding and stressful.^1, 7^

Doctors in general work longer hours than most other workers and Table 1 confirms that the majority of GPs in this sample work more than the average 40-hour week. Other authors have alluded to increased stress in doctors who work longer hours.^1^ This seems to confirm that if doctors spend most of their time at work, with less time devoted to other activities such as socialising, family, sport and hobbies, they are at an increased risk of work-related stress and burnout.^9^ Thus GPs who spend long hours at their practice and have few compartments^13^ to their life seem to be more likely to suffer from work-related stress.

Large patient numbers per day (median 30 patients and a maximum of 60 patients per day) will mean that very little time is spent on each consultation and that there will be no opportunity for the doctor to take any time off during his or her busy day. The quality of the consultations, which are less than 10 minutes per patient, may also be drastically impaired in the practice where the GP sees 60 patients per day.

The results of the GHQ-12 indicate that stress among Kwa-Dukuza GPs (10 or 38% stressed according to Table 4) is slightly higher than found in other studies that indicate a prevalence of 28% among doctors.^5^ Six doctors were severely stressed and these doctors may need psychological intervention and are at a high risk for burnout. However, since this study was totally anonymous, these doctors cannot be identified.

The majority of the responding GPs feel they are stressed (22 from Table 2). Of these, eight doctors were stressed as measured by the GHQ-12 (see Table 5). Nearly all the doctors who responded perceive themselves to be stressed at work. This may mean that they are unhappy at work but not objectively stressed as yet. This probably implies that at present they can cope with their working situation and still feel that they are playing a valuable role at work. These doctors may be at risk of work-related stress and would benefit from stress-relieving and coping strategies before they reach burnout or become totally disillusioned with working as a doctor.

The results indicate that GPs practising in a small town are just as susceptible to stress as GPs elsewhere. The results may imply that GPs need to be aware of their risk of work-related problems and stress and need to concentrate on ways to reduce their stress. This would involve primarily having other interests or compartments to their lives, as suggested by Couper^3^ and Gaede.^9^

### Conclusion

The doctors of Kwa-Dukuza would benefit from this study by becoming aware of their high-levels of work-related stress. Those doctors who have a perception of being stressed and are not yet objectively stressed would benefit most from intervention centring on methods of stress reduction and coping mechanisms. Strategies to reduce stress should be implemented among these GPs. The majority of doctors of Kwa-Dukuza would benefit from workshops or other training in techniques, especially aimed at GPs as part of the caring profession, that may be used to relieve work-related stress. Doctors should also take cognisance of Couper's^3^ suggestion that there must be other compartments/areas of interest in their lives besides medicine. This cross-sectional survey in Kwa-Dukuza confirms that work-related stress among doctors is high and that doctors need to take active measures to reduce their stress.
